# Rs864745 in *JAZF1*, an Islet Function Associated Variant, Correlates With Plasma Lipid Levels in Both Type 1 and Type 2 Diabetes Status, but Not Healthy Subjects

**DOI:** 10.3389/fendo.2022.898893

**Published:** 2022-07-01

**Authors:** Hao Dai, Yu Qian, Hui Lv, Liying Jiang, Hemin Jiang, Min Shen, Heng Chen, Yang Chen, Shuai Zheng, Qi Fu, Tao Yang, Kuanfeng Xu

**Affiliations:** Department of Endocrinology and Metabolism, The First Affiliated Hospital of Nanjing Medical University, Nanjing, China

**Keywords:** *JAZF1*, variant, diabetes, islet function, dyslipidemia

## Abstract

**Objective:**

This study aims to reveal the association between *JAZF1* rs864745 A>G variant and type 2 diabetes (T2D), type 1 diabetes (T1D) risk, and their correlation with clinical features, including islet function, islet autoimmunity, and plasma lipid levels.

**Methods:**

We included 2505 healthy controls based on oral glucose tolerance test (OGTT), 1736 unrelated T2D, and 1003 unrelated autoantibody-positive T1D individuals. Binary logistic regression was performed to evaluate the relationships between rs864745 in *JAZF1* and T2D, T1D, and islet-specific autoantibody status under the additive model, while multiple linear regression was used to assess its effect on glycemic-related quantitative traits and plasma lipid levels.

**Results:**

We did not find any association between rs864745 in *JAZF1* and T2D, T1D, or their subgroups (All P > 0.05). For glycemic traits, we found that the G allele of this variant was significantly associated with higher 120 min insulin level, insulinogenic index (IGI), corrected insulin response (CIR), and acute insulin response (BIGTT-AIR) (P = 0.033, 0.006, 0.009, and 0.016, respectively) in healthy individuals. Similar associations were observed in newly diagnosed T2D but not T1D individuals. Although this variant had no impact on islet autoimmunity (All P > 0.05), significant associations with plasma total cholesterol (TC) and low-density lipoprotein (LDL) level stratified by *JAZF1* rs864745 variant were observed in the disease status of T2D (P = 0.002 and 0.003) and T1D (P = 0.024 and 0.009), with significant heterogeneity to healthy individuals.

**Conclusions:**

The common *JAZF1* rs864745 variant contributes to islet function and lipid metabolism, which might be put into genetic risk scores to assess the risk of related clinical features.

## Introduction

*JAZF* Zinc Finger 1 (*JAZF1*), a multifunctional regulatory factor, was initially identified as an orphan nuclear receptor corepressor and involved in gluconeogenesis and lipid metabolism. The human *JAZF1* gene, located on chromosome 7p15.2, is predominantly expressed in insulin-responsive organs such as the liver, fat, skeletal muscle, and pancreas (1, 2). Furthermore, *JAZF1* is expressed in both human (3, 4) and mouse β-cells (5, 6). *JAZF1* is an important regulator of endoplasmic reticulum (ER) stress and ribosome biogenesis *via* a feedback action preventing the activation of ER and p53 stress-mediated β-cell apoptosis (7), which plays a pivotal role in the differentiation of β-cells and glucose homeostasis (8). Obesity (9) and diabetes status (10, 11) alter the *JAZF1* expression pattern.

Genome-wide association studies (GWAS) have shown that the risk A allele of rs864745, an intron variant in *JAZF1*, correlates with both type 1 (T1D) (12) in Europeans, and type 2 diabetes (T2D) (13) in various ethnicities, including Europeans, Americans, as well as Asians (11, 14–17). Moreover, this variant also correlated with β-cell function (14, 18).

Studies have demonstrated that T1D and T2D have heterogeneous characteristics in terms of both genetic (13, 19) and phenotypic features (20, 21). Previous studies have not fully elucidated the underlying contribution of this variant on T1D and T2D subtypes and related clinical phenotypes. Therefore, this study aimed to shed light on the relationship between rs864745 in *JAZF1* and T1D, T2D subgroups, and also β-cell function and lipid metabolism in both healthy and diabetes disease status.

## Materials and Methods

### Study Population

This study includes unrelated 1736 T2D, 1003 T1D, and 2505 non-diabetic controls. Their clinical characteristics are shown in **Table 1.** All the unrelated healthy controls and T2D subjects were recruited from Nanjing, one of the 25 communities in the REACTION study (22), and unrelated T1D participants were recruited from the First Affiliated Hospital of Nanjing Medical University between January 2008 and December 2020. T2D and T1D were diagnosed according to the World Health Organization criteria, and diabetes caused by liver dysfunction, pancreatitis, or other autoimmune diseases was excluded. Furthermore, T1D cases were identified as only diabetic patients with at least one autoantibody-positive [zinc transporter 8 antibody (ZnT8A), glutamate decarboxylase antibody (GADA), or insulinoma-associated-2 antibody (IA-2A)]. Non-diabetic controls were extracted based on oral glucose tolerance test (OGTT), and the inclusion criteria for healthy controls were as follows: HbA1c ≤ 6.0% (4.2 mmol/mol), fasting plasma glucose < 6.1 mmol/L, and two-hour plasma glucose < 7.8 mmol/L. The study population was determined as Chinese Han by questionnaire. All samples were collected with appropriate informed consent from all participants and/or their guardians in a written way. This study was approved by the Ethics Committee from the First Affiliated Hospital of Nanjing Medical University and conducted according to the Declaration of Helsinki II principles.

**Table 1:** Overall study population and metabolic index characteristics.

	T2D	T1D	Control
n	1736	1003	2505
Sex (male/female)	750/986	491/512	786/1719
Age (years)	62.0 ± 8.0	23.7 ± 14.8	54.0 ± 9.0
BMI (kg/m^2^)	25.20 ± 3.47	19.84 ± 3.78	23.65 ± 3.00
Lipids medication (%)	3.1%	–	1.0%
**Plasma glucose (mmol/l)**			
Fasting	7.66 ± 2.27	–	5.33 ± 0.36
30 min post OGTT	11.26 ± 3.06	–	8.58 ± 1.48
120 min post OGTT	12.79 ± 4.17	–	6.14 ± 0.98
**Serum insulin (mIU/L)**			
Fasting	11.32 (8.29, 16.25)	–	9.86 (7.38, 13.38)
30 min post OGTT	23.62 (14.51, 41.79)	–	57.13 (37.80, 88.59)
120 min post OGTT	43.07 (22.74, 83.70)	–	40.72 (26.25, 63.37)
HbA1c (%)	7.10 ± 1.50	11.15 ± 3.06	5.60 ± 0.40
HDL (mmol/L)	1.23 ± 0.30	1.36 ± 0.61	1.37 ± 0.34
LDL (mmol/L)	2.83 ± 0.81	2.69 ± 1.03	2.81 ± 0.74
TC (mmol/L)	4.86 ± 1.06	4.31 ± 1.84	4.88 ± 0.96
TG (mmol/L)	1.82 ± 1.32	1.52 ± 1.91	1.37 ± 0.85
**Serum antibody positive rate**			
ZnT8A	–	0.45	–
GADA	–	0.74	–
IA-2A	–	0.50	–

Data are expressed as mean ± SD or as median (interquartile range).

T2D, type 2 diabetes; T1D, type 1 diabetes; BMI, body mass index; OGTT, oral glucose tolerance test; HbA1c, glycated hemoglobin; HDL, high-density lipoprotein; LDL, low-density lipoprotein; TC, total cholesterol; TG, triglyceride; ZnT8A, zinc transporter 8 antibody; GADA, glutamate decarboxylase antibody; IA-2A, insulinoma-associated-2 antibody.

### Laboratory Measurements for Glycemic and Lipid Traits and Islet-Specific Autoantibodies

All non-diabetic controls and T2D individuals measured plasma glucose and serum insulin levels at fasting, 30, and 120 min after OGTT. Serum C-peptide levels were measured in newly diagnosed T1D patients (disease duration less than 3 months) on fasting, 30, 60, 120, and 180 min standard mixed meal tolerance test (MMTT). Serum insulin levels were measured by an insulin radioimmunoassay kit (BNIBT, China). Serum C-peptide levels and plasma lipid levels, including high-density lipoprotein cholesterol (HDL), low-density lipoprotein cholesterol (LDL), total cholesterol (TC), and triglyceride (TG), were determined by the chemiluminescence method (Roche Diagnostics, Switzerland). The methods for detecting islet-specific autoantibodies were described in our previous studies (19).

### Genotyping Assay

Genomic DNA was extracted from peripheral blood lymphocytes using the QIAamp DNA Blood Extraction Kit (QIAGEN, Germany). Genotyping of the *JAZF1* rs864745 variant was performed using the Sequenom Massarray (BGI CO.LTD, China). The primer sequences and UEP primers were as follows: Forward, 5-ACGTTGGATGCATTGAACATTTCCTACAACC-3; Reverse, 5-ACGTTGGATGCCATATAAGTGATGCTCAAA-3; UEP, 5-ATGCTCAAATATAATTTGAACTGTTA-3. The genotyping success rate was above 98%, and 100 randomly selected samples were repeated for complete agreement. The genotype distribution in control, T2D, and T1D groups were consistent with Hardy-Weinberg equilibrium (P > 0.05).

### Statistical Analyses

We measured and calculated insulin release and sensitivity derived from the OGTT in non-diabetic controls and T2D individuals according to the formulas described previously, including homeostasis model assessment of β-cell function (HOMA-B) and insulin resistance (HOMA-IR), insulinogenic index (IGI), acute insulin response index (BIGTT-AIR), insulin sensitivity index (BIGTT-SI), corrected insulin response (CIR), and Matsuda’s insulin sensitivity index (Matsuda ISI) (23). Serum insulin, C-peptide levels, insulin release and resistance index were log-transformed, and then statistically analyzed. Under an appropriately adjusted additive model, a binary logistic regression analysis was performed for the relationship between rs864745 in *JAZF1* and T2D and T1D subgroups, and islet-specific autoantibody status. The effect of this variant on glycemic quantitative traits and serum lipid levels were analyzed by multiple linear regression. The glycemic quantitative traits were adjusted by sex, age, and BMI except that BIGTT-AIR and BIGTT-SI were corrected for age. Serum lipid levels were also adjusted by lipids medication in addition to age, gender, and BMI. All P-values were two-sided, and P < 0.05 was considered significant. The heterogeneity was considered significant with *I^2^* > 75% and P < 0.05. Statistical analyses were performed using SPSS 18.0 and STATA 11.0.

## Results

### *JAZF1* rs864745 A>G Variant Is Not Associated With the Risk of Either T2D or T1D Subgroups

As shown in **Table 2**, we did not find any association between *JAZF1* rs864745 A>G variant and T2D or T1D risk in total (P > 0.05). Although obesity is a critical risk factor for T2D, no association with this variant was observed in lean or obese T2D subgroups (P > 0.05), stratified by BMI status according to the Chinese criteria (24). As age at diagnosis is an important marker for classifying different subgroups of T1D (21), we divided the T1D population into two subgroups according to age at diagnosis ≥ 18 and < 18 years old, and the results revealed no significant association in either children/adolescents or adults T1D subgroups (P > 0.05).

**Table 2:** The association between the *JAZF1* rs864745 A>G variant and T1D, T2D risk.

Groups	Genotype distribution			ORadj (95% CI)	P
	AA	AG	GG		
**T2D**					
**Total**	1020	619	97	0.972 (0.870, 1.086)	0.616
**BMI < 24kg/m** ^2^	372	228	35	0.958 (0.808, 1.136)	0.620
**24 ≤ BMI < 28kg/m** ^2^	464	296	46	0.978 (0.845, 1.130)	0.760
**BMI ≥ 28kg/m** ^2^	184	95	16	0.838 (0.620, 1.133)	0.250
**T1D**					
**Total**	589	360	44	0.930 (0.739, 1.171)	0.538
**Diagnosed age ≥ 18**	358	196	18	0.922 (0.730, 1.166)	0.498
**Diagnosed age < 18**	228	157	24	0.613 (0.262, 1.435)	0.259
**Control**	1454	896	155		

A/G: risk/non-risk alleles for T1D, T2D (ref 11–18). G allele is the effect allele for Oradj. T2D: Binary logistic regression, dependent variable: whether diabetic or not, independent variables: genotype, sex, age. BMI normal < 24kg/m^2^, overweight ≥ 24kg/m^2^, < 28kg/m^2^, obese ≥ 28kg/m^2^. T1D: Binary logistic regression, dependent variable: whether diabetic or not, correction: sex, age (non-onset age). A total of 12 T1D patients have unknown diagnosed age. T2D, type 2 diabetes; T1D, type 1 diabetes; BMI, body mass index.

### *JAZF1* rs864745 A>G Variant Significantly Correlates With OGTT-Related Insulin Release, but Not Insulin Sensitivity Indices in Healthy Individuals

We analyzed OGTT-derived indicators of glycemic-related traits in healthy individuals. As shown in **Table 3**, this variant did not affect fasting, 30 min, or 120 min plasma glucose levels. However, we found that the G allele of this variant significantly improved islet function associated with 120 min insulin level, IGI, CIR, and BIGTT-AIR (P = 0.033, 0.006, 0.009, and 0.016, respectively). We further stratified them by BMI status, the G allele of this variant was significantly associated with high IGI, BIGTT-AIR (P = 0.01 and 0.037, respectively) in normal-weight (BMI < 24kg/m^2^) individuals, as shown in **Table S1**. However, we did not find any association of this variant with insulin release or insulin sensitivity in obese or overweight individuals. Subsequently, we also assessed the impact of this variant on the islet function of newly diagnosed T2D and T1D individuals. This variant was associated with 120 min insulin level in newly diagnosed T2D individuals (p = 0.041), as shown in **Table S2**. However, this variant did not affect fasting or responsive C-peptide levels in newly diagnosed T1D individuals, as shown in **Table S3**. We also evaluated the association with islet-specific autoantibody status, but we did not find any relationship to the positive rate of ZnT8A (p = 0.723), GADA (p = 0.101), or IA-2A (p = 0.316), as shown in **Table 4**.

**Table 3:** The association of the *JAZF1* rs864745 A>G variant with glycemic quantitative traits in healthy individuals based on OGTT.

Control	AA	AG	GG	β	SE	Padj
**Plasma glucose (mmol/l)**						
Fasting	5.33 (5.10, 5.58)	5.32 (5.05, 5.58)	5.34 (4.95, 5.62)	-0.029	0.011	0.144
30 min post OGTT	8.57 (7.65, 9.59)	8.43 (7.58, 9.46)	8.43 (7.49, 9.30)	-0.037	0.047	0.057
120 min post OGTT	6.21 (5.49, 6.88)	6.16 (5.56, 6.85)	6.38 (5.58, 7.00)	0.015	0.031	0.433
**Serum insulin (mIU/L)**						
Fasting	9.83 (7.36, 13.22)	9.93 (7.45, 13.65)	10.27 (7.27, 13.64)	0.021	0.007	0.273
30 min post OGTT	56.14 (37.34, 85.83)	57.49 (37.44, 92.70)	61.30 (43.99, 85.60)	0.034	0.009	0.078
120 min post OGTT	39.52 (26.12, 61.27)	41.93 (26.28, 63.88)	43.81 (27.66, 73.57)	0.041	0.010	0.033*
**Islet function**						
HOMA-B	105.72 (80.78, 151.27)	110.29 (81.39, 154.66)	117.08 (80.98, 159.25)	0.036	0.008	0.072
IGI	14.77 (8.64, 24.91)	16.09 (9.06, 28.59)	16.67 (8.70, 27.75)	0.054	0.013	0.006**
BIGTT-AIR	7.58 (7.29, 7.94)	7.61 (7.34, 8.04)	7.68 (7.37, 8.00)	0.048	0.009	0.016*
CIR	149.66 (90.35, 238.67)	163.72 (96.68, 274.66)	168.27 (94.78, 270.96)	0.051	0.011	0.009**
**Insulin resistance**						
HOMA-IR	2.31 (1.71, 3.12)	2.35 (1.75, 3.26)	2.45 (1.67, 3.35)	0.016	0.007	0.423
Matsuda ISI	0.07 (0.04, 0.12)	0.07 (0.04, 0.11)	0.06 (0.04, 0.11)	-0.033	0.010	0.080
BIGTT-SI	1.94 (1.54, 2.24)	1.92 (1.50, 2.25)	1.88 (1.48, 2.16)	-0.024	0.009	0.224
**HbA1c%**	5.60 (5.40, 5.90)	5.70 (5.40, 5.90)	5.60 (5.40, 5.90)	0.004	0.012	0.826

Linear regression, with several correction indices calculated separately, with genotype and correction indices as independent variables and islet function as dependent variable. *P < 0.05; **P < 0.01.

OGTT, oral glucose tolerance test; HOMA-B, homeostasis model assessment of β-cell function; IGI, insulinogenic index; BIGTT-AIR, acute insulin response; CIR, corrected insulin response; HOMA-IR, homeostasis model assessment of insulin resistance; Matsuda ISI, Matsuda’s insulin sensitivity index; BIGTT-SI, insulin sensitivity index; HbA1c, glycated hemoglobin.

**Table 4:** The association between the *JAZF1* rs864745 A>G variant and T1D risk stratified by islet autoantibody status.

	Pos	Neg	OR_adj_ (95% CI)	*P_adj_*
**ZnT8A**				
AA	265	307		
AG	161	190	0.960(0.767-1.202)	0.723
GG	17	24		
**GADA**				
AA	426	161		
AG	276	84	1.240(0.959-1.603)	0.101
GG	36	8		
**IA-2A**				
AA	280	308		
AG	186	173	1.122(0.896-1.404)	0.316
GG	23	21		

For each autoantibody, association tests included sex, genotype, age at T1D diagnosis, and disease duration as covariates. Numbers in parentheses indicate the frequency of that genotype. P < 0.05 was considered significant.

ZnT8A, zinc transporter 8 antibody; GADA, glutamate decarboxylase antibody; IA-2A, insulinoma-associated-2 antibody.

### Significant Associations With TC and LDL Levels Stratified by the *JAZF1* rs864745 Variant Are Observed in T2D and T1D Status, With a Significant Heterogeneity Among Healthy Individuals

We did not find any association between this variant and plasma lipid levels (HDL, LDL, TC, TG) in the healthy individuals based on OGTT (All P > 0.05). However, the G allele of this variant was associated with lower plasma LDL and TC levels in both T2D (P = 0.003 and 0.002) and T1D status (P = 0.009 and 0.024). Compared with healthy controls, their impacts on LDL and TC levels had significant heterogeneity in T2D (P = 0.049 and 0.030) and T1D (P = 0.017 and 0.034) status, as shown in **Table 5**.

**Table 5:** Correlation of plasma TC and LDL in people with different metabolic states and stratified by *JAZF1* rs864745 variant.

Groups	AA	AG	GG	β	SE	adjusted P	P_het_	*I^2^* (%)
**Control**								
HDL	1.37 ± 0.34	1.39 ± 0.34	1.36 ± 0.34	0.006	0.010	0.728		
LDL	2.81 ± 0.73	2.81 ± 0.78	2.75 ± 0.63	-0.016	0.024	0.431		
TC	4.88 ± 0.96	4.89 ± 0.98	4.81 ± 0.82	-0.011	0.031	0.588		
TG	1.36 ± 0.85	1.36 ± 0.82	1.49 ± 0.95	0.021	0.027	0.273		
**T2D**								
HDL	1.24 ± 0.30	1.21 ± 0.29	1.22 ± 0.26	-0.043	0.011	0.061	0.093	64.60
LDL	2.87 ± 0.82	2.77 ± 0.81	2.67 ± 0.75	-0.071	0.032	0.003**	0.049*	75.70
TC	4.92 ± 1.08	4.80 ± 1.05	4.62 ± 0.96	-0.072	0.042	0.002**	0.030*	78.70
TG	1.84 ± 1.36	1.85 ± 1.33	1.50 ± 0.71	-0.030	0.052	0.204	0.109	61.10
**T1D**								
HDL	1.36 ± 0.71	1.34 ± 0.39	1.75 ± 0.32	0.061	0.058	0.188	0.228	31.30
LDL	2.78 ± 1.08	2.53 ± 0.94	2.49 ± 0.91	-0.138	0.097	0.009**	0.017*	82.50
TC	4.46 ± 2.00	4.09 ± 1.49	3.72 ± 1.94	-0.103	0.143	0.024*	0.034*	77.70
TG	1.61 ± 2.16	1.35 ± 1.40	1.74 ± 1.63	-0.061	0.605	0.185	0.169	47.00

Linear regression analysis was used, with lipid index as the dependent variable and age, sex, lipids medication and BMI (type 1 not used) as independent variables. All P values were two-tailed and P < 0.05 was considered as significant. *P < 0.05; **P < 0.01.

T2D, type 2 diabetes; T1D, type 1 diabetes; HDL, high-density lipoprotein; LDL, low-density lipoprotein; TC, total cholesterol; TG, triglyceride.

## Discussion

Previous studies have found a shared genetic risk factor underlying T1D and T2D etiology (25, 26), with approximately 60 chromosome regions associated with T1D (27) and over 200 associated with T2D (28), reaching genome-wide significance. Therefore, uncovering co-localizing signals could provide biological insights into overlapping disease mechanisms, and potentially reveal therapeutic targets effective for both diseases. The *JAZF1* region was reported as the genome-wide significant region in T1D (12) and T2D (13). However, the results were inconsistent. For instance, several studies revealed an association between the *JAZF1* rs864745 variant and T2D, whereas others did not find any association (11, 13–17, 29–32). Our study supported that the *JAZF1* rs864745 variant did not associate with T1D, T2D, or their subgroups, at least in the Chinese population.

Although obesity is a critical risk factor for the heterogeneity of T2D, and patients with different BMI have various natural histories, and β-cell mass (33), no association with this variant was reported in T2D subgroups stratified by BMI level in this study. Meanwhile, age at diagnosis is a critical factor for T1D subgroups (21). Our previous studies also confirmed a genetic correlation among T1D subgroups of diagnosed ages (19, 34). Nevertheless, our current study shows that the *JAZF1* rs864745 variant is not associated with the onset age of T1D. Explanations for these discrepancies may include differences in sample size, environment, and populations studied. Because minor genetic variation accumulates over time, ancestral groups that became geographically separated many generations ago may yield different genetic risks. Additionally, the subjects in our study included community residents over 40 years old Chinese subjects, and aged participants are expected to have more cumulative environmental exposure and thus be more likely to be affected by gene-environment interaction.

Further studies have suggested that the T allele of rs864745 was associated with abnormal pancreatic β-cell function (14), which plays a role in the pathogenesis of T2D (17). *JAZF1* rs864745 T allele is linked to reduced *JAZF1* mRNA expression and decreased insulin release (14). Furthermore, an autosomal genomic scan showed that the *JAZF1* rs864745 T variant is associated with increased fasting insulin levels (14). Our results are consistent with those of the prior studies showing that the G allele of this variant mainly improves β-cell function in normal glucose tolerance (NGT) and newly diagnosed T2D subjects, especially in those NGT participants with normal BMI levels. However, no effects on β-cell function or insulin sensitivity were observed in the overweight or obese subgroups. We speculate that the impact of the variant on islet β-cell function might be inferior to metabolic impact in people with excess body weight. These indicate that when translating genetic risk loci into clinical features of the disease state, the effect of a single variant is insufficient to achieve significant changes. As a result, multi-genic risk loci should be loaded into the estimation in future studies.

Besides, previous GWAS studies have revealed the genetic associations with islet autoantibody positivity in T1D subjects (35, 36). We evaluated the association with islet-specific autoantibody status with this variant, but no significant relationship to the positive rate of ZnT8A, GADA, or IA-2A was observed. Additionally, this variant did not affect fasting or responsive C-peptide levels in newly diagnosed T1D patients in our study. These findings indicated that the *JAZF1* rs864745 A>G variant might influence islet β-cell function, but not islet autoimmunity. Further validation is warranted considering the sample size and selected population in our study.

Studies have reported that dyslipidemia is a well-established risk factor for T1D and T2D (37–39). *JAZF1* is a metabolic regulator to improve lipid metabolism and resist hyperglycemia through multiple metabolic signaling pathways in T2D (18). The beneficial effects of *JAZF1* on lipid metabolism have been observed in hepatocytes and adipocytes (18, 40–42). In the liver, *JAZF1* enhances fatty acid β-oxidation. In adipocytes, *JAZF1* inhibits the accumulation of fatty acids and triglycerides. A previous multi-ancestry population-based study has revealed multiple variants associated with serum lipid traits (43). Of these, JAZF1 rs864745 A>G variant correlates with TG and HDL. Since the non-diabetic controls recruited in our study were a subgroup of the total population, this locus was not significantly associated with HDL, LDL, TC, or TG in our normoglycemic individuals. However, we found that the G allele of this variant was associated with lower plasma LDL and TC levels in both T2D and T1D status, suggesting the relationship may be more significant during high glucose pathological states. The present findings might carry significant clinical implications. Moreover, databases from GTEx (https://gtexportal.org/), demonstrated that this variant affected *JAZF1* gene expression in multi tissues, including the liver, skeletal muscle, and pancreas. Thus, we speculated that two distinct mechanisms for this variant are involved in protecting individuals from diabetes. One is by improving β-cell function, and the other is modulating lipid metabolism (via decreasing TC and LDL levels) in the hyperglycemia state.

In conclusion, we found that the *JAZF*1 rs864745 variant is linked to the improvement of β-cell function as well as lipid metabolism. Considering the limitation of the reproducibility with a single variant, more T1D or T2D genetic risk loci should be further studied for clinical subgroups and phenotypic of T1D and T2D patients in diabetes screening and precision therapy.

## Data Availability Statement

The original contributions presented in the study are included in the article/**Supplementary Material**, further inquiries can be directed to the corresponding authors.

## Ethics Statement

The studies involving human participants were reviewed and approved by the Ethics Committee from the First Affiliated Hospital of Nanjing Medical University. Written informed consent to participate in this study was provided by the participants’ legal guardian/next of kin.

## Author Contributions

HD performed statistical analyses and interpretation of data and drafted the initial manuscript. HL, YQ, and LJ were responsible for the analyses and interpretation of data. HJ, MS, and HC contributed to laboratory measurements. YC and SZ contributed to the collection and selection of samples. QF and TY gave a critical revision of the manuscript. KX directed the study design and provided a critical revision of the manuscript. All the co-authors gave the final approval of the version.

## Funding

This study was supported by grants from the National Natural Science Foundation of China (81670715, 81830023, 82070803 and 81900708), and the Young Scholars Fostering Fund of the First Affiliated Hospital of Nanjing Medical University (PY2021005).

## Conflict of Interest

The authors declare that the research was conducted in the absence of any commercial or financial relationships that could be construed as a potential conflict of interest.

## Publisher’s Note

All claims expressed in this article are solely those of the authors and do not necessarily represent those of their affiliated organizations, or those of the publisher, the editors and the reviewers. Any product that may be evaluated in this article, or claim that may be made by its manufacturer, is not guaranteed or endorsed by the publisher.

## Abbreviations

T1D, type 1 diabetes; T2D, type 2 diabetes; NGT, normal glucose tolerance; TC, total cholesterol; LDL, low-density lipoprotein; HDL, high-density lipoprotein; TG, triglyceride; *JAZF1*, juxtaposed with another zinc finger gene 1; GWAS, genome-wide association studies; OGTT, oral glucose tolerance test; MMTT, mixed meal tolerance test; BMI, body mass index; HbA1c, glycated hemoglobin; ZnT8A, zinc transporter 8 antibody; GADA, glutamate decarboxylase antibody; IA-2A, insulinoma-associated-2 antibody; HOMA-B, homeostasis model assessment of β-cell function; HOMA-IR, homeostasis model assessment of insulin resistance; IGI, insulinogenic index; BIGTT-AIR, acute insulin response; BIGTT-SI, insulin sensitivity index; CIR, corrected insulin response; Matsuda ISI, Matsuda’s insulin sensitivity index.
