# Supplementary material for: Rs864745 in JAZF1, an Islet Function Associated Variant, Correlates With Plasma Lipid Levels in Both Type 1 and Type 2 Diabetes Status, but Not Healthy Subjects
Source: Front Endocrinol (Lausanne). 2022 Jul 1;13:898893. doi: 10.3389/fendo.2022.898893 (PMC9283698; doi:10.3389/fendo.2022.898893)
Supplement: Supplementary file 1 [file DataSheet_1.docx]

**Table S1 Relationship between *JAZF1* rs864745 A>G variants and islet function in healthy controls**

|  | **AA** | **AG** | | **GG** | **β** | **SE** | ***P*** |
| --- | --- | --- | --- | --- | --- | --- | --- |
| **BMI < 24kg/m^2^** |  |  | |  |  |  |  |
| **Plasma glucose (mmol/l)** |  |  | |  |  |  |  |
| Fasting | 5.29 (5.07, 5.56) | 5.29 (5.02, 5.54) | | 5.31 (4.95, 5.63) | -0.019 | 0.016 | 0.479 |
| 30 min post OGTT | 8.46 (7.52, 9.53) | 8.36 (7.37, 9.41) | | 8.43 (7.48, 9.34) | -0.015 | 0.066 | 0.584 |
| 120 min post OGTT | 6.04 (5.39, 6.75) | 6.07 (5.47, 6.70) | | 6.33 (5.39, 6.99) | -0.027 | 0.043 | 0.304 |
| **Serum insulin ( mIU/L)** |  |  | |  |  |  |  |
| Fasting | 9.12 (6.89, 11.77) | 9.34 (6.83, 12.50) | | 9.20 (6.77, 13.40) | 0.021 | 0.010 | 0.432 |
| 30 min post OGTT | 50.23 (34.09, 76.56) | 51.40 (33.78, 82.37) | | 58.97 (41.72, 82.17) | 0.047 | 0.012 | 0.076 |
| 120 min post OGTT | 35.69 (23.87, 52.34) | 37.88 (24.10, 56.91) | | 40.38 (24.88, 69.78) | 0.050 | 0.013 | 0.053 |
| **Islet function** |  |  | |  |  |  |  |
| HOMA-B | 101.19 (76.63, 142.96) | 103.89 (78,23, 148.51) | | 111.92 (79.09, 152.16) | 0.032 | 0.010 | 0.217 |
| IGI | 13.28 (7.48, 23.11) | 14.38 (8.00, 25.70) | | 16.07 (7.72, 28.57) | 0.067 | 0.018 | 0.010* |
| BIGTT-AIR | 7.45 (7.23, 7.76) | 7.48 (7.25, 7.84) | | 7.57 (7.27, 7.90) | 0.055 | 0.011 | 0.037* |
| CIR | 133.69 (82.62, 223.30) | 146.11 (86.95, 250.02) | | 146.76 (85.60, 270.53) | 0.048 | 0.048 | 0.062 |
| **Insulin resistance** |  |  | |  |  |  |  |
| HOMA-IR | 2.15 (1.61, 2.83) | 2.20 (1.56, 2.96) | | 2.17 (1.56, 3.34) | 0.017 | 0.010 | 0.523 |
| Matsuda ISI | 0.09 (0.05, 0.13) | 0.08 (0.05, 0.13) | | 0.07 (0.04, 0.13) | -0.043 | 0.014 | 0.100 |
| BIGTT-SI | 2.13 (1.81, 2.35) | 2.09 (1.77, 2.36) | | 1.94 (1.66, 2.31) | -0.038 | 0.011 | 0.145 |
| **24 ≤ BMI < 28kg/m^2^** |  |  |  | |  |  |  |
| **Plasma glucose (mmol/l)** |  |  |  | |  |  |  |
| Fasting | 5.38 (5.12, 5.61) | 5.37 (5.11, 5.59) | 5.41 (4.93, 5.66) | | -0.004 | 0.019 | 0.899 |
| 30 min post OGTT | 8.65 (7.70, 9.65) | 8.48 (7.74, 9.42) | 8.28 (7.41, 9.21) | | 0.045 | 0.080 | 0.186 |
| 120 min post OGTT | 6.38 (5.63, 6.97) | 6.38 (5.65, 7.03) | 6.38 (5.70, 6.90) | | 0.064 | 0.054 | 0.058 |
| **Serum insulin ( mIU/L)** |  |  |  | |  |  |  |
| Fasting | 10.35 (7.63, 14.12) | 10.74 (8.25, 14.35) | 10.75 (8.28, 14.23) | | 0.049 | 0.012 | 0.151 |
| 30 min post OGTT | 64.68 (41.95, 98.94) | 63.23 (43.21, 103.40) | 60.6 (45.18, 85.88) | | 0.030 | 0.016 | 0.377 |
| 120 min post OGTT | 44.52 (29.08, 71.36) | 48.43 (30.05, 71.67) | 44.19 (28.35, 81.13) | | 0.036 | 0.016 | 0.286 |
| **islet function** |  |  |  | |  |  |  |
| HOMA-B | 110.66 (82.22, 155.43) | 114.69 (89.69, 159.09) | 120.33 (86.25, 160.33) | | 0.061 | 0.014 | 0.071 |
| IGI | 17.00 (10.06, 28.97) | 18.25 (10.53, 31.29) | 16.63 (9.76, 24.56) | | 0.026 | 0.021 | 0.447 |
| BIGTT-AIR | 7.71 (7.48, 8.10) | 7.77 (7.49, 8.13) | 7.75 (7.46, 8.08) | | 0.040 | 0.017 | 0.235 |
| CIR | 168.96 (99.84, 272.26) | 176.48 (107.41, 285.03) | 178.71 (102.19, 248.37) | | 0.055 | 0.019 | 0.103 |
| **insulin resistance** |  |  |  | |  |  |  |
| HOMA-IR | 2.49 (1.81, 3.30) | 2.54 (1.93, 3.50) | 2.54 (1.87, 3.42) | | 0.041 | 0.012 | 0.229 |
| Matsuda ISI | 0.06 (0.04, 0.10) | 0.06 (0.04, 0.09) | 0.06 (0.04, 0.10) | | -0.040 | 0.017 | 0.237 |
| BIGTT-SI | 1.77 (1.33, 2.05) | 1.72 (1.37, 2.00) | 1.69 (1.30, 2.00) | | -0.020 | 0.015 | 0.559 |
| **BMI ≥ 28kg/m^2^** |  |  |  | |  |  |  |
| **Plasma glucose (mmol/l)** |  |  |  | |  |  |  |
| Fasting | 5.47 (5.19, 5.75) | 5.47 (5.14, 5.72) | 5.35 (5.13, 5.55) | | -0.087 | 0.041 | 0.227 |
| 30 min post OGTT | 9.02 (8.25, 9.76) | 8.86 (7.89, 9.89) | 8.60 (7.64, 9.48) | | 0.034 | 0.157 | 0.638 |
| 120 min post OGTT | 6.64 (5.75, 7.23) | 6.46 (5.92, 6.94) | 6.69 (5.93, 7.19) | | 0.032 | 0.101 | 0.657 |
| **Serum insulin ( mIU/L)** |  |  |  | |  |  |  |
| Fasting | 13.35 (10.13, 38.31) | 13.14 (9.76, 18.19) | 11.94 (9.11, 13.41) | | -0.128 | 0.023 | 0.073 |
| 30 min post OGTT | 83.78 (59.08, 117.38) | 95.74 (61.24, 148.30) | 78.74 (50.91, 85.56) | | -0.054 | 0.032 | 0.454 |
| 120 min post OGTT | 58.47 (38.63, 90.55) | 52.41 (38.02, 74.21) | 59.23 (35.36, 81.34) | | -0.044 | 0.030 | 0.538 |
| **Islet function** |  |  |  | |  |  |  |
| HOMA-B | 138.58 (104.65, 182.31) | 142.98 (102.65, 191.96) | 115.53 (98.92, 183.98) | | -0.097 | 0.024 | 0.174 |
| IGI | 18.14 (12.84, 26.99) | 22.63 (14.80, 39.78) | 21.31 (12.94, 27.59) | | 0.050 | 0.041 | 0.483 |
| CIR | 187.28 (119.61, 265.73) | 235.23 (141.64, 340.66) | 212.67 (110.62, 282.9) | | -0.014 | 0.055 | 0.844 |
| **Insulin resistance** |  |  |  | |  |  |  |
| HOMA-IR | 3.18 (2.40, 4.26) | 3.27 (2.40, 4.37) | 2.72 (1.98, 3.14) | | -0.135 | 0.023 | 0.060 |
| Matsuda ISI | 0.04 (0.03, 0.06) | 0.04 (0.03, 0.06) | 0.05 (0.04, 0.08) | | 0.112 | 0.031 | 0.119 |

**Note:** Linear regression, several indicators were calculated separately, with genotype and corrected indicators as independent variables and islet function as dependent variable. Normal < 24kg/m^2^, overweight ≥ 24kg/m^2^, <28kg/m^2^, obese ≥ 28kg/m^2^. *P < 0.05. Abbreviations: BMI, body mass index; OGTT, oral glucose tolerance test; HOMA-B, homeostasis model assessment of beta cell function; IGI, insulinogenic index; BIGTT-AIR, acute insulin response; CIR, corrected insulin response; HOMA-IR, homeostasis model assessment of insulin resistance; Matsuda ISI, Matsuda’s insulin sensitivity index; BIGTT-SI, insulin sensitivity index.

**Table S2 Relationship between JAZF1 rs864745 A>G variants and indicators of islet function in newly-diagnosed T2D individuals**

|  | **AA** | | **AG** | **GG** | **β** | **SE** | **Padj** |
| --- | --- | --- | --- | --- | --- | --- | --- |
| **Plasma glucose (mmol/l)** | |  |  |  |  |  |  |
| Fasting | 7.12 (6.25, 8.29) | | 7.15 (6.30, 8.04) | 6.71 (6.07, 7.71) | -0.007 | 0.121 | 0.862 |
| 30 min post OGTT | 10.94 (9.11, 12.81) | | 10.98 (9.29, 12.84) | 10.99 (9.78, 12.93) | -0.002 | 0.170 | 0.948 |
| 120 min post OGTT | 12.35 (10.10, 15.00) | | 12.34 (10.07, 14.55) | 12.05 (10.55, 14.09) | -0.030 | 0.236 | 0.434 |
| **Serum insulin ( mIU/L)** |  | |  |  |  |  |  |
| Fasting | 11.21 (8.01, 16.17) | | 11.34 (8.41, 16.12) | 12.19 (9.08, 16.73) | 0.014 | 0.014 | 0.700 |
| 30 min post OGTT | 22.92 (14.31, 40.83) | | 24.31 (14.51, 44.50) | 28.28 (17.60, 44.01) | 0.044 | 0.019 | 0.225 |
| 120 min post OGTT | 42.17 (23.10, 81.50) | | 43.57 (21.70, 86.37) | 55.24 (28.41, 105.60) | 0.075 | 0.020 | 0.041* |
| **Islet function** |  | |  |  |  |  |  |
| HOMA-B | 63.44 (41.48, 95.05) | | 63.06 (39.49, 98.34) | 74.44 (50.20, 107.19) | 0.015 | 0.018 | 0.697 |
| IGI | 3.42 (1.26, 7.91) | | 3.66 (1.48, 8.27) | 3.88 (1.14, 7.20) | 0.024 | 0.029 | 0.511 |
| BIGTT-AIR | 6.98 (6.54, 7.33) | | 6.96 (6.57, 7.29) | 7.11 (6.62, 7.37) | 0.015 | 0.023 | 0.691 |
| CIR | 34.21 (18.78, 63.61) | | 35.56 (19.23, 61.94) | 40.68 (22.54, 71.05) | 0.030 | 0.025 | 0.420 |
| **Insulin resistance** |  | |  |  |  |  |  |
| HOMA-IR | 3.68 (2.49, 5.65) | | 3.68 (2.64, 5.53) | 3.73 (2.66, 5.95) | 0.010 | 0.016 | 0.786 |
| Matsuda ISI | 0.05 (0.03, 0.09) | | 0.05 (0.02, 0.09) | 0.04 (0.03, 0.06) | -0.060 | 0.020 | 0.090 |
| BIGTT-SI | 1.25 (0.62, 1.71) | | 1.21 (0.65, 1.71) | 1.19 (0.45, 1,57) | -0.078 | 0.025 | 0.042* |

**Note**: Linear regression, with several correction indices calculated separately, with genotype and correction indices as independent variables and islet function as dependent variable.*P < 0.05. Abbreviations: T2D, type 2 diabetes; OGTT, oral glucose tolerance test; HOMA-B, homeostasis model assessment of beta cell function; IGI, insulinogenic index; BIGTT-AIR, acute insulin response; CIR, corrected insulin response; HOMA-IR, homeostasis model assessment of insulin resistance; Matsuda ISI, Matsuda’s insulin sensitivity index; BIGTT-SI, insulin sensitivity index.

**Table S3 Association of *JAZF1* rs864745 A>G variant with C-peptide levels in newly-diagnosed T1D subjects**

| **C-peptide** | **AA** | **AG** | **GG** | **β** | **SE** | **Padj** |
| --- | --- | --- | --- | --- | --- | --- |
| C0 | 117.10 (44.85, 236.70) | 118.94 (33.30, 227.83) | 103.23(58.00,180.00) | 0.026 | 19.322 | 0.661 |
| C30 | 250.40 (86.69, 435.10) | 186.48 (55.44, 413.30) | 168.90 (109.98, 309.68) | -0.054 | 43.112 | 0.498 |
| C60 | 306.36 (115.70, 511.90) | 254.79 (46.62, 487.68) | 216.80 (93.24, 241.30) | -0.017 | 56.160 | 0.814 |
| C120 | 320.15 (84.23, 623.73) | 283.05 (86.58, 567.50) | 301.50 (240.11, 479.00) | -0.013 | 51.820 | 0.835 |
| C180 | 423.70 (173.08, 817.23) | 479.00 (98.07, 805.60) | 396.40 (258.32, 656.90) | 0.099 | 82.000 | 0.220 |

**Note:** These associations were calculated using linear regression analysis with an additive model, screening individuals with T1D of less than three month duration, adjusted for sex and age at diagnosis of T1D. Abbreviations: T1D, type 1 diabetes.
